# Supplementary material for: Are carbon emissions trading and green financial instruments synergistic? -Comprehensive quantitative research based on content analysis
Source: PLoS One. 2024 Mar 7;19(3):e0298601. doi: 10.1371/journal.pone.0298601 (PMC10919601; doi:10.1371/journal.pone.0298601)
Supplement: S1 Data — (ZIP) [file pone.0298601.s001.zip › Data/地级市Z_F工作报告.zip!/╣π╢1⁄2/╢1⁄2▌╕╩╨/└┤╫╘╣1⁄2╓┌║┼í╛┬φ┐╦╩2╛▌═°í┐_2013.docx]

各位代表：

现在，我代表市人民政府向大会报告2012年政府工作，对2013年工作提出建议，请予审议。并请政协各位委员和其他列席人员提出意见。

2012年工作回顾

刚刚过去的2012年，是东莞承前启后、加快转型的关键一年。一年来，在上级和市委的坚强领导下，全市各级各部门以科学发展观为指导，围绕市第十三次党代会确立的“加快转型升级、建设幸福东莞、实现高水平崛起”的战略目标，按照优化发展、好中求进的方针，从容应对复杂严峻的发展形势，经济增长稳步回升，社会保持和谐稳定，市第十五届人大一次会议确定的年度工作任务基本完成。预计全市实现生产总值5010亿元，同比增长6.1%。人均生产总值6万元，增长4.9%。来源于东莞的财政收入845.6亿元，其中市公共财政预算收入356.3亿元，增长13.8%。进出口总额增长6.8%左右，出口增长8.5%左右。城市居民人均可支配收入42872元，农民人均纯收入24898元，分别增长8.5%和9%。

过去一年，是高水平崛起谋篇开局、凝心聚力的一年。紧紧围绕市委决策部署，提质提效提精神，强力强势抓执行。大力推进“六个东莞”建设，紧盯“三重一大”建设、科技金融产业融合、水乡地区统筹发展、镇村集体经济转型等突破口，出台实施一系列新举措，抓出了工作实效，实现了良好开局。

过去一年，是创新型经济扬帆起步、坚实推进的一年。一批关系城市未来的战略性、创新型产业项目纷纷落户，众多著名高校院所与创新团队竞相进驻，科技孵化和风投创投机构迅速增长，为新一轮产业大发展积蓄了较大后劲。东莞在《福布斯》中文杂志大陆创新能力最强城市的评比中名列第十三位。

过去一年，是城市形象不断提升、工作亮点纷呈的一年。生产总值突破五千亿元大关，工业投资、服务业吸引外资逆势增长，财政收入、利用外资等多项指标位居全省前列。中国加工贸易产品博览会作为我市首次举办的国家级重大展会，为东莞增添了一张亮丽的城市名片。全省加工贸易转型升级现场会、世界莞商大会、中国图书馆年会等重大活动成功举办，“三打两建”、广深高速沿线景观整治等专项行动强力实施，商事登记、行政审批等重大改革锐意推进，全国文明城市、全国双拥模范城市、中国制造业最优投资环境城市、中国十佳宜业城市等荣誉称号的蝉联获取，进一步彰显了东莞创新活力的经济形象、宜居生态的城市形象、安定和谐的社会形象。

一年来，我们主要抓了以下工作：

——突出推进“三重一大”建设，以产业增量扩张促进存量调整。集全市之力开展重大项目招商。制定实施“1+5”政策，实行“一站通”工作机制，开展日韩台招商活动，赴以色列、德国进行经贸考察，到京拜访央企总部，落实土地资源保障，引进了投资超600亿元的粤海装备技术产业园、123亿元的中粮集团粮油食品加工园区、100亿元的华为终端总部等项目。全市共引进重大项目81宗，投资总额达1972亿元，为下一阶段发展注入了新的强大动力。扎实推进重大平台和重大项目建设。松山湖启动中以国际科技合作产业园、两岸生物技术产业合作基地的建设，台湾高科技园升格为省级平台。虎门港成功跨入百万标箱港口行列。生态园基础设施建设加快。长安新区用海规划通过国家评审。市属重大项目完成投资278.3亿元，增长18.6%，带动全社会固定资产投资1178亿元，增长9.1%。切实加快产业转型升级步伐。推进外经贸“十个100”计划，全年新增来料加工转法人企业536家，外资企业新设研发机构220家，总数达到670家。外资企业产品内销总额增长15.1%，省级以上名牌名标达580个。主营业务超百亿元企业增至3家。促进信息化和工业化融合，省两化融合“4个100”示范工程标杆企业增至37家。大力发展现代服务业，社会消费品零售总额增长9.2%，服务业增加值占生产总值比重超过50%。

——力促科技、金融与产业融合，增强经济创新驱动力。

积极实施招科引智战略。组团拜访商务部、科技部、教育部等部委及北大、中科院等高校院所，达成50多项合作意向。与北大共建光电研究院，与华南理工共建协同创新研究院，与中科院共建云计算中心，推进散裂中子源项目建设。全市公共创新平台增至13家。启动松山湖大学创新城规划建设。引进省创新科研团队5个，总数达14家，排名全省第三。科技合作周暨招才引智大会升格为部省共同举办。完善激励创新政策体系。制定实施科技金融产业融合“1+4”政策，完善“科技东莞”政策，引导更多社会资金投入科技创新。全市R&D占生产总值的比重提高到1.58%。先进制造业、高技术制造业增加值分别占规模以上工业增加值的42.7%和32.3%。专利申请量、授权量位居全省第三。发挥金融创新引领作用。设立20亿元创业投资引导基金，成功引进深创投、中科招商等企业，风投创投等股权投资类企业增至98家。上市企业增至12家。加快金融商务区规划，推进松山湖科技金融产业融合试点。新设和引进金融机构15家，跨境人民币结算突破1000亿元。我市成为粤台金融合作试点城市，台湾玉山银行东莞分行正式开业。

——加大水乡统筹与集体经济转型力度，促进区域协调发展。作出统筹水乡地区发展的重大决策。出台实施方案，全面铺开十镇一港规划编制工作，启动了龙湾滨江片区、水乡大道改造提升工程等12个总投资额307亿元的先期项目。水乡发展已被纳入省幸福导向型产业体系行动计划。推进镇村集体经济改革发展。出台了一系列含金量高、操作性强的政策，加大力度破解集体经济发展难题。开展清产核资，建成集体资产交易试点平台，严控一般性支出和超前分配。镇街平均可支配收入8.3亿元；村组两级纯收入增长9.4%，其中欠发达村纯收入增长15%。镇村债务负担有所减轻，收不抵支的村（社区）减少34个。强化深莞惠等区域合作与对接。深莞惠规划衔接、产业融合、交通对接等日益加强，石马河联合整治取得阶段性成效。主动加强穗莞战略合作。加快莞韶、莞惠产业转移园建设。认真抓好援疆援藏工作，圆满完成对韶关、云浮的帮扶开发“双到”任务，对口援助广西河池、重庆巫山工作扎实推进。与韩国牙山市结为友好城市。

——打造法治化国际化营商环境，增创政府服务“加一”和综合成本“减一”优势。力推商事登记和行政审批制度改革。在大朗进行商事登记制度改革试点，企业注册时间平均缩短60%以上，市场主体同比增长79.3%，成为全省发证最快、同期发证最多的试点地区。去年12月份起在全市铺开该项改革，市本级不再保留任何涉及市场准入的前置审批事项。东莞被国家工商总局确定为全国商事登记改革试点城市。大力深化行政审批制度改革，第一批取消和转移行政审批事项296项，减幅41.3%。积极帮助企业减负和解决实际问题。开展市镇领导“访企业、送服务、促转型”大走访。全面取消个体工商户和工商企业治安联防费，减半收取流动人员调配费，为企业年减负3.8亿元。海关、检验检疫等口岸单位主动为企业减免收费近2亿元。进一步规范镇村收费。实施新10亿元融资支持计划。深入开展“三打两建”行动。以罕见力度严查大要案、斩断利益链、深挖保护伞，加快建设社会信用体系和市场监管体系。累计查办欺行霸市、制假售假及商业贿赂案件3万多宗，查处保护伞233人，促进形成风清气正的政务环境、公平诚信的市场秩序。不断创新提升政府服务。在全国率先推出加工贸易管理服务平台，实现了外经贸、海关、检验检疫和企业“四方联网”。推进依法行政，开展“市民评机关”活动，对服务窗口进行明查暗访，着力解决推诿扯皮、办事拖拉、效率低下等问题，优化了政府服务，提高了行政效率。

——深入推进城乡环境整治，提升城市建设管理水平。高效完成广深高速、虎门大桥东莞段景观综合整治。用短短五个月时间，清理广告标牌1333块，清拆违法和临时建筑44.7万平方米，沿线脏乱差的面貌有较大改观。以对城市未来发展高度负责的态度，强力开展在建违法建筑清理专项行动。加快轨道交通等重大基础建设。组建东实集团，推进轨道交通融资。地铁R2线工程进展顺利，3个站点完成主体封顶。新火车站等15项重点工程竣工，市民艺术中心等21项重点工程加快推进。新建改造高速路、国省道、镇村联网路147.7公里，建成一批水电气管网和水利防灾减灾工程。加快“三旧”改造步伐。新增和更新公交车、出租车近千辆。加强环境生态保护。划定27个重点饮用水源保护区，启动水源保护隔离工程。推进生态景观林带和森林公园建设，植树造林2226公顷，新建绿道324公里。城镇生活垃圾无害化处理率达85.2%。深化运河综合整治，落实节能减排任务，加强环境执法和污染企业监管。

——加强社会建设与管理创新，着力保障和改善民生。积极创建全省创新社会管理引领区。成立社会建设研究院。完成社会组织孵化基地建设。出台政府购买服务目录。稳步推进村级体制改革。加强网上信访大厅建设，全省率先实现视频信访系统镇街全覆盖。建立全市统一的劳动关系预警系统。强化综治信访维稳平台建设，做好矛盾隐患排查化解，引导民众理性表达个人意愿与爱国诉求。切实强化社会安全管理。推进“四化五警”建设，开展警务前移、出租屋治安管理等试点，推广执法办案格式化，启动智能综合视频监控系统建设，“两抢一盗”立案下降9.6%，老虎机赌博现象基本绝迹。严格落实安全生产“一岗双责”，推进消防安全网格化管理试点，深入开展校车隐患等专项整治行动，强化食品药品监管，全市未发生重特大安全事故。大力发展以改善民生为重点的社会事业。市财政全年共投入205亿元用于民生事业。基本完成向社会承诺的十件实事。落实幼儿园财政补助，上调镇街中小学公用经费标准。全市高考录取率、每万户籍人口升重点人数等四项主要高考指标均居全省第一。深入实施提升公共文化服务水平工程，健全文化产业、文化精品扶持政策，一批优秀文艺作品获省级以上重大奖项。成功举办漫博会。出台公立医院改革试点实施方案，全面启用市中医院新院。建设十分钟体育圈，莞籍运动员首获残奥会金牌。发行新社保卡，上调离退休人员基本养老金和居家养老服务补助标准。加强劳动力培训和就业援助。向困难群众发放临时救助金和物价补贴。国防人防、统计审计、人口计生、外事侨务、工青妇幼、民族宗教、档案方志、科普法普、气象、打私等工作扎实推进。

各位代表！一年来，全市上下凝心聚力，提振精神，以强烈的使命感克难前行，以高效的执行力扎实推进，朝着高水平崛起的道路迈出了坚实的步伐。在此，我代表市人民政府，向全市广大干部群众，向人大代表和政协委员，向各民主党派、人民团体、社会各界人士，向各驻莞单位、驻莞部队，以及参与、支持和关心东莞建设发展的港澳台同胞和海外侨胞，致以崇高的敬意和衷心的感谢！

与此同时，我们也清醒地看到，东莞还处于转型攻坚期和矛盾凸显期，资源环境瓶颈约束加剧、产业结构不尽合理、自主创新能力不强、体制机制不够完善等深层次问题仍然存在，需要长期不懈努力破解。从当前来看，也有不少困难和问题值得我们高度重视：一是经济企稳回升的基础仍需巩固。尽管全市经济呈现出回暖向好的态势，但由于受国际经济环境影响，生产总值、工业、消费等指标与年度目标任务仍有一定距离。企业生产经营也面临着不少困难。二是重大项目的带动效应尚未真正显现。一大批“三重”项目虽已签约启动，但要真正落地动工、建成投产、发挥效益，还需付出艰辛努力。三是统筹发展的力度有待进一步加大。集体经济转型困难不小，区域发展不够协调，镇村债务负担仍然较重，水乡地区统筹发展等工作仍需深入推进，对土地资源的整合力度亟待加大。四是城市规划建设管理的水平有待进一步提升。城市顶层设计相对缺乏，经营管理需要创新理念，违法建筑等问题不容忽视。五是惠及民生的工作仍待加大力度。十件实事涉及的52项具体工作中，有2项未能完成年度目标。群众对物价上涨、公交出行、人才入户等问题反映依然较多，治安管理、教育公平、医疗改革等任务比较艰巨。六是机关作风建设仍需加强。一些干部的精神状态与高水平崛起的要求仍有一定的差距，办事效率、服务水平和管理能力有待进一步提高。对这些问题，我们必须以强烈的责任感和紧迫感，积极寻求破解之策。

2013年工作安排

今年是全面贯彻落实十八大精神的开局之年，也是东莞加快推动高水平崛起的奋进之年，机遇与挑战并存。世界经济正在经历新一轮的再平衡过程，低速增长态势仍将延续，总需求不足和产能相对过剩的矛盾有所上升，企业经营成本增加和创新能力不足的问题并存，经济发展和资源环境硬约束的矛盾有所加剧。与此同时，党的十八大胜利召开为今后发展指明了方向，跨国公司和大型央企民企酝酿着新一轮生产布局调整，第三次工业革命雏形初现，国内城镇化、工业化、信息化融合步伐不断加快，新兴产业和重大项目进驻东莞势头强劲，为我市经济稳健回暖与产业加速升级提供了良好契机。今年政府工作的总体要求是：认真贯彻落实党的十八大、中央经济工作会议、省委十一届二次全会和市委十三届三次全会精神，以习近平总书记视察广东和胡春华书记莅莞调研重要讲话精神为指导，深入实践科学发展观，坚定不移地推动转型升级，脚踏实地推进改革发展，把握好稳中求进的主基调，以提高经济增长质量和效益为中心，以打造创新型经济为主攻方向，着力优环境、上项目、强统筹、抓改革，为加快转型升级、建设幸福东莞、实现高水平崛起不断开创新局面。

具体来讲，优环境，就是要加大力度降低综合成本，提升政府服务，提高城市承载力，推动东莞成为综合营商环境最优越的城市之一。上项目，就是要坚定不移地抓产业、盯项目，全力谋求重大项目招商与建设的新突破，加快推进经济结构战略性调整。强统筹，就是要按照主体功能定位和产业转型升级的要求，下更大力气推动水乡统筹、土地统筹、招商统筹，以此为突破口优化资源配置，促进协调发展，提升发展层次。抓改革，就是要以自我革命的勇气和决心，大刀阔斧地推进行政体制、社会管理等重点领域改革，坚决破除思想观念和体制机制约束，不断增创新的制度红利。

综合考虑各方面因素，今年全市发展的预期目标是：生产总值增长7%，人均生产总值增长6%，市公共财政预算收入增长10%，固定资产投资总额增长10%，社会消费品零售总额增长10%，出口总额增长7%；服务业增加值占生产总值比重50%，先进制造业、高技术制造业增加值占规模以上工业增加值比重分别达到42.5%和33.8%，发明专利申请量和授权量分别增长15%和60%；城市居民人均可支配收入增长8.5%，农民人均纯收入增长9%，单位生产总值能耗下降4.86%，城镇登记失业率、居民消费价格涨幅分别控制在3%以内和3%左右。

围绕以上目标要求，我们必须坚持加快转型升级、推进发展方式转变不动摇，矢志不渝地把转型升级作为东莞践行科学发展观的核心任务，继续加大产业结构调整力度，以“工作落实年”为主题，切实抓好转型升级各项重大决策的贯彻落实，抓好重大项目的跟踪落实，抓好市委1号文和市政府1号文等政策措施的细化落实。重点做好几方面工作：

一、打造最佳营商环境，进一步提升开放型经济水平

争做综合营商成本最具竞争力的城市。进一步清理和规范涉企收费，全面放开再生资源回收市场，建立统一交易平台，全年为企业减轻行政收费、中介服务收费、中间环节成本35亿元以上。办好一年一度的加博会，发挥台博会、漫博会等平台作用，推广“一达通”、“大麦客”等商业模式，探索规划和申报综合保税区，增加车检场保税物流相关功能，推进省市共建物联网产业基地，大力发展现代流通业，帮助企业降低渠道成本和物流成本。落实领导走访、专人跟进和跨部门服务等机制，开展竞争性高峰电力电量交易试点，帮助企业解决用工、用电、融资等困难以及物业产权等历史遗留问题。巩固“三打两建”成果，加大打击力度，总结石龙镇社会信用体系建设、食品行业市场监管体系建设等试点经验，在全市铺开“两建”工作。

增强城市对优秀人才的吸引力。全面梳理和改革人才入户政策，进一步降低门槛和简化程序。大力引进创新科研团队、领军人才、紧缺人才和高技能人才。妥善解决人才住房问题，出台企业人才子女入学办法，做好家属安置工作，营造有利于吸引和留住人才的软硬环境。推进留学人员创业园、博士后工作站等载体建设。打造大学生入户快速通道。

实施创新驱动发展战略。加快松山湖大学创新城等平台建设，发挥现有平台作用，促进与专业镇和产业集群对接。用好财政引导资金，继续引进风投创投机构，加大金融创新和企业上市培育力度。扶持高端电子、生物技术、新一代互联网、3D打印等战略性新兴产业发展，与省共建云计算应用产业基地。实施高成长型中小企业培育计划，支持中小微企业和民营经济发展。积极创建全国质量强市示范城市。办好科技合作周活动。

大力发展电子商务。出台扶持电子商务发展的政策措施。支持专业镇和专业市场搭建电子商务营销配送网络，带动产业集群整合升级，助推传统产业向价值链高端延伸。深入开展“莞货网上行”等活动。加力引进和培育电子商务企业，对在莞设立总部的电商予以奖励。探索实行“零门槛”的电子商务企业网上注册登记制度，吸引各地电商在莞集聚。

深化加工贸易转型与内外开放合作。用好国家赋予的先行先试政策，鼓励加工贸易企业外包供应链管理，向研发设计、品牌营销、物流配送转型。抓好外贸转型升级示范基地建设，扩大设备及先进技术进口，提高一般贸易占比，促进对外贸易多元平衡发展。推动两岸生物技术产业合作基地建设，用好专项投资基金，铺开基础设施及首期项目。推进粤台金融合作试点。加快台湾高科技园详细性规划编制和招商工作，推进中以国际科技合作产业园建设。落实珠三角规划纲要，加强深莞惠在规划、交通、产业等方面的对接，推进穗莞和莞港澳合作。

二、深入实施“揽月行动”，构建“星月同辉”的产业格局

建立统筹有力的招商机制。认真落实“1+5”招商政策，建立重大项目招商信息共享、布局统筹、选址流转和利益分享机制，将镇街、园区和部门跟踪接洽的重大项目全部纳入统一数据库，避免过度竞争和无序引进。建立项目评估准入、比选和约束机制，制定投资强度、单位产出、产业链配套等项目评价体系，健全企业失信惩戒和享受优惠政策的退出机制，强化对重大项目招商工作的指导。

以高效服务推动粤海等重大项目动工建设。进一步强化与粤海集团的战略合作，尽快完成粤海园区概念性总体规划和产业规划，力争项目于今年动工。加快启动“三纵三横”配套路网一期工程建设。推动华为终端总部项目年内动工。对所有重大项目实行挂钩督导、部门联审、“六个一”包干等制度，提供从立项审批到办证开工“一条龙”服务。

科学从容推进园区开发建设。完善松山湖功能配套，严格筛选项目，提升产出效益，争创国家创新型科技园区。加快生态园征地拆迁和土地平整，完善产业规划和招商指引，积极申报国家城市湿地公园。加快长安新区围填海工程与基础设施建设。深化虎门港与沙田镇统筹发展，突出产业链招商，推进集装箱码头和保税物流中心建设，做大增值服务，做旺港口经济。

争创全国城乡土地生态利用制度综合改革试点城市，为产业发展进一步拓展空间。推进土地统筹整合，优化国土空间开发格局，未来三年力争腾挪拼接出1000亩以上地块50块、面积10万亩，强化重大项目用地保障。探索实行土地规划发展权和开发建设权分离，试行闲置土地置换、项目分期供地、土地绩效评估等制度。落实土地未开发收回政策。加快“三旧”改造步伐。创建全国节约集约用地模范市。对新增用地指标实行分类比例控制和差别化管理。

三、强力统筹水乡地区十镇一港发展，打造全省幸福导向型产业发展示范区

建立健全规划体系和统筹开发机制。高标准编制水乡地区统筹发展总体规划和专项规划，一盘棋考虑城乡建设、生态保护和产业布局。加快完善投融资、利益平衡、项目把关等机制，强化统筹发展的制度保障。推动农村管理体制、土地统筹机制等改革在水乡地区先行先试。

完善一体化的水乡交通体系。市财政安排20亿元，加快水乡大道、疏港大道粤晖大桥、望牛墩横海大桥、石龙红海大桥等先期项目建设，迅速启动中洪路、望万路等干线联网工程。统筹推进基础设施和公共服务设施建设。探索道路建设与统筹开发沿线土地相结合的城建投融资新模式。

打造具有水乡特色的标志性片区。提前启动望洪枢纽站周边15平方公里土地统筹和建设，致力打造东莞城市次中心、水乡核心片区。加快建设总投资超百亿元的龙湾滨江片区，促进商住休闲旅游综合开发。加快推进中堂、望牛墩10.5平方公里的水乡风情区规划，实行整体招商，成片开发。加快建设麻涌4平方公里世界农场项目，打造集都市农业、观光旅游、创意休闲等于一体的新型农场。启动挂影洲中心涌沿线片区更新改造，促进污染治理、环境美化与产业发展有机结合。

逐步构建亲水宜居的优质生活圈。通过生物净化、湿地生态改造等手段，积极推进水环境治理，改善水体质量和生态景观。实施城乡环境综合治理行动，不断改善村容村貌，加强土地管理，集约节约利用土地，提高土地利用效率。推进亲水环境建设，打造具有岭南水乡特色的区域景观。

四、力推重点领域综合改革，增创新的制度红利

东莞改革开放30多年来的成功经验，很关键的一条就是以敢为人先的精神和改革创新的锐气，不断推进体制机制上的众多首创改革，从而适应了市场经济发展的要求，破解了瓶颈制约，赢得了发展先机。我们要拿出前几代东莞人破釜沉舟、筚路蓝缕的勇气，向改革要方法、要动力，冲破束缚发展的思想观念和路径依赖，冲破利益固化的藩篱，攻坚克难，在转型升级上杀出一条新的血路，形成各项改革协同推进的正能量。

以商事登记为突破口深化行政体制改革。按照宽进严管原则，完善商事登记改革配套政策，推行网上年检，强化后续监管，优化后续审批，进一步发挥商事登记改革的综合影响力，推动与商事主体改革相关的其他改革向纵深发展。完成行政审批制度改革，落实配套工作，推行并联审批、网上审批和“一站式”服务。今后未经市政府批准一概不许增设审批事项。适时扩大简政强镇改革范围。探索推进大部门体制改革。

推进农村综合改革和转型发展。全面推进农村综合改革各项任务。市镇财政加大投入，逐步统筹承接村一级的治安、环卫和行政管理开支。推进社区政务服务中心建设。完善和落实欠发达镇村扶持政策。通过税收分成、生态补偿、结对帮扶等途径，让基层分享土地统筹、“三重”建设等带来的实惠。引导基层通过集中统租、稳妥投资等方式，实现集体资产保值增值。

推进省市共建全省创新社会管理引领区。深化警务机制改革，在现有基础上再推动公安市局10%、分局20%的警力下沉。整合全市治安联防队伍。加力整治治安重点场所和黄赌毒现象，严打各类犯罪活动。落实安全生产“一岗双责”，抓好危险化学品、消防、交通安全等专项整治，创新食品药品和农产品安全监管模式，推进应急管理综合一期平台建设，落实领导接访下访、信访包案等制度，打造平安东莞。培育和规范社会组织发展，新建10个市级社区综合服务中心示范点。

抓好医疗、公交、水务等领域的改革。按照公益化方向加快公立医院改革步伐，组建市属公立医院管理中心。启动公交公益化改革，提升服务质量和管理水平，整治公交不准点、冒黑烟及车内治安等群众关注的问题。深化水资源管理体制机制改革，推进镇街水务一体化，实现市镇村三级水务分级管理。

五、切实加强经济调节，夯实可持续发展的制度保障

加强财政资金及财政投资项目的绩效管理。全面强化财政专项资金、财政投资重大基建、重大民生支出和政府融资项目的绩效评价，进一步提高财政资金使用效益。建立绩效评价结果反馈与应用机制，实行评价结果与预算安排相结合。加强审计监督。严肃查处非法骗取财政扶持资金的行为。

进一步规范镇村经济管理。建立农村集体资产交易平台和“三资”管理信息监控平台。加强对大额开支和敏感性开支的监控，严控非生产性支出。强化债权债务管理，推进镇村已到期债务清偿。今后凡镇村借债，必须经上一级政府批准。

完善与国有资产规模相适应的监管机制。全面调查摸底和分类梳理，建立健全科学合理的人事、审计、工资、业绩考核等监管机制，强化国资管理部门对市属国有企业的监督管理。做优做强做活国有企业，推动国有资本更多地投向关系经济命脉的行业和领域。摸清财政投资建设的场馆、医院、学校等国有资产底数，探索构建新型监管模式。

六、提升城市承载力，建设更具生态文明的美丽东莞

优化城市顶层设计。以新一轮总体规划修编为契机，构建疏密有致的城市格局。以轨道交通建设为纽带，加强沿线土地统筹开发，拓展和优化城市空间布局。加快南城国际商务区、中央商圈、生态休闲区和松山湖大道两侧区域的规划建设，提高城市首位度。推进从莞高速东莞段、东江梨川大桥、松山湖大道延长线等建设，完善城市基础网络和配套功能。

加强环境综合治理。推广广深高速景观综合整治经验，切实加强全市公路沿路景观和广告标牌管理。推进四大环保行动计划，加快环保产业基地建设，创建国家生态市。加强东江水保护，深化运河与内河涌整治，加快截污次支管网铺设。加快垃圾处理厂建设，整治垃圾填埋场，推广垃圾分类处理。严格落实节能减排责任，抓好国家级工业能耗在线监测试点工作。逐步淘汰黄标车。大力植树造林，加快森林公园、镇村公园、慢行道等建设，提高绿道使用效益。深入创建宜居社区。

提升城市精细化管理水平。建设“智慧城市”和“数字城管”，在大市区率先推行网格化管理，将绿化环卫、城市照明、户外广告等纳入监管范围。全面清理在建违法建筑，完善长效管理机制。推进“智慧交通”建设，大力整治交通拥堵，加快主要堵塞点的改造和分流。统筹市政地下管线铺设，在新开发区域推广管线集中统一铺设的共同沟建设。加强工程招投标管理，保证市场公平与工程质量。

创新投融资平台建设。发挥好新组建的东莞实业等投融资平台作用，强化建设及监督管理，有效整合多种资源，创新投融资机制，撬动更多社会资本参与城市建设和发展。重点抓好轨道交通融资，探索通过发行市政债券、利用股权信托资金等方式，筹集资金加快建设。

七、在改善民生中加强社会建设，努力让市民过上美好生活

完善保障有力的社保体系。认真办好十件实事。加大就业创业扶持力度，重点解决大学毕业生、困难家庭等群体的就业问题。提高最低生活保障和五保供养标准。扩建市社会福利中心，将居家养老服务扩大至120个社区。发行300万张新社保卡，加大重大疾病和意外伤害医疗保障力度。加强平价商店和价格调节基金运营管理，减少物价上涨对群众的影响。

增强文化整体实力和竞争力。全面推进文化名城建设，继续实施“百千万文化惠民”工程，建好国家公共文化服务体系示范区。加大文化产业和文化精品扶持力度，办好“我们的节日”、读书节等活动，加强非物质遗产和历史文物的保护与开发利用，实施文化人才“三个100”工程。

努力办好群众满意的教育。新建5所公办幼儿园，给予民办学校在校生公用经费和教科书补助。增加新莞人子女积分入学学位。统一全市初中公用经费标准，加强民办学校规范化建设，推进免费中职教育。启用东莞理工学校新校区、技师学院和高技能公共实训中心，开设市外国语学校，扩建市特殊教育学校。加强在莞高校学科建设，提高服务地方发展能力。

全面发展体育卫生人口计生等各项事业。大力发展群众体育和竞技体育，增加社区体育设施，提升篮球、羽毛球等品牌。推进市人民医院分院、儿童医院、台心医院等建设。发挥好社区卫生服务机构的作用，为市民提供优质价廉的医疗服务。探索设立市国医馆，推进中医药进社区。实施优生健康惠民工程和妇女两癌免费筛查。稳步扩大住房公积金制度覆盖面。落实好新疆兵团农三师的对口支援计划，推进支援西藏林芝的项目建设。继续帮扶韶关、云浮、广西河池、重庆巫山等地发展。

八、进一步加强作风建设，塑造高效廉洁的政府形象

落实中央“八项规定”，深入改进工作作风。精简会议和文件，改进会风文风，力戒形式主义、文牍主义。严格控制各类庆典、多重检查、评比及达标创建活动。加强调查研究，推广现场办公。开展明查暗访，落实首问责任、限时办结等制度。加强政府执行力建设，着力治庸治懒治散治奢，整治“吃拿卡要”等行为，推动机关作风进一步好转。

推进“大数据”战略，加快电子政务建设。运用云技术集成企业登记、信用监管、数据统计等功能，构建全市信息共享和内部监管平台。拓展加工贸易“四方联网”，打造地方电子口岸。优化提升网上办事平台，推动90%的行政审批事项、80%的公共服务事项上网，打造全国一流的网上办事大厅。推进数据开放工程，在财政、环保、招投标等领域开展数据公开试点。

强化内外监督，打造为民务实清廉的政府。落实廉洁从政各项规定和廉政责任制，强化政府内部监督，坚决惩治腐败行为。加快法治政府建设，落实重大行政决策程序和政务公开制度，主动接受人大政协及社会监督，重视和发挥网络媒体监督作用，促进形成清廉高效、务实为民的良好政风。

各位代表！九万里风鹏正举，党的十八大绘就了夺取中国特色社会主义新胜利的宏伟蓝图，中华民族伟大复兴的目标已越来越接近，得改革开放风气之先的东莞没有理由不认真把握这一千载难逢的发展机遇。站在新的起跑线上，我们将牢记使命，勤勉进取，在中共东莞市委的坚强领导下，与全市人民一道，同心同德，攻坚克难，朝着高水平崛起的目标奋勇前进，用实干托起我们的东莞梦和伟大的中国梦！
